# Supplementary material for: TIM3 Checkpoint Inhibition Fails to Prolong Survival in Ovarian Cancer-Bearing Mice
Source: Cancers (Basel). 2024 Mar 14;16(6):1147. doi: 10.3390/cancers16061147 (PMC10969025; doi:10.3390/cancers16061147)
Supplement: Supplementary file 1 [file cancers-16-01147-s001.zip › cancers-2820789-supplementary.pdf]

TIM3 checkpoint inhibition fails to prolong survival in ovarian cancer bearing mice

Berckmans Y.[1], *Vankerckhoven A.*[1], *Caro Audhut A.*[1,2,3], *Kempeneers J.*[1,4], *Thirion G.*[1], *Vandenbrande K.*[1], *Lanickova T.*[5] , *Fucikova J.*[5,6], *Vergote I.*[4], *Laoui D.*[2,3] ,*Coosemans A.*[1]

[1]*Laboratory of Tumor Immunology and Immunotherapy, Department of Oncology, Leuven Cancer Institute, KU Leuven, Leuven, Belgium.*
[2]*Laboratory of Dendritic Cell Biology and Cancer Immunotherapy, VIB Center for Inflammation Research, 1050 Brussels, Belgium.*
[3]*Laboratory of Cellular and Molecular Immunology, Vrije Universiteit Brussel, 1050 Brussels, Belgium*
[4]*Department of Gynaecology and Obstetrics, Leuven Cancer Institute, University Hospitals Leuven, Belgium*
[5]*Sotio Biotech, Prague, Czech Republic.*
[6]*Department of Immunology, Charles University, 2nd Faculty of Medicine and University Hospital Motol, Prague, Czech Republic*

Supplementals TIM3 paper

Supplementary Table S1: Overview of studies included after literature search, ordered by subject

Publication	Publication type	Population	Samples	(Immune) cells studied	% positivity
**TIM3**					
Sawada *et al.* (2020)	Paper	100 ovarian cancer patients	Tumour biopsy, PBMCs	CD8+ T cells	22.8%
Li *et al.* (2018)	Paper	22 ovarian cancer patients	Tumour biopsy, PBMCs	CD4+CXCR5+ Tfh cells	69.3%
Bu *et al.* (2016)	Paper	25 ovarian cancer patients	Tumour biopsy, PBMCs	CD3+CD4+CD25+ Treg cells, CD3+CD8+ T cells	-
Wu *et al.* (2013)	Paper	52 ovarian cancer patients	PBMCs	CD4+ T cells, CD8+ T cells	CD4+ T-cells: 4.89%<br>CD8+ T-cells: 2.91%
Guo *et al.* (2013)	Paper	Murine model	Peritoneal immune cells	CD4+ T cells, CD8+ T cells, CD4+FoxP3+ Treg cells, CD11b+Gr-1+ MDSC	-
Yan *et al.* (2013)	Paper	Not mentioned	Tumour biopsy, PBMCs	Tumor infiltrating lymphocytes, peripheral blood lymphocytes	-
Hinchcliff *et al.* (2018)	Paper	44 recurrent ovarian cancer patients	-	-	-
Curigliano *et al.* (2019)	Conference abstract	Not mentioned	-	-	-
Laeremans *et al.* (2018)	Conference abstract	30 ovarian cancer patients	Tumour biopsy	-	-
Eiva *et al.* (2017)	Conference abstract	-	-	CD137+ TIL	-
Wong *et al.* (2016)	Conference abstract	-	PBMCs	Macrophages	-

Lee *et al.* (2015)	Conference abstract	41 ovarian cancer patients	PBMCs	Tregs, MDSC, exhausted CD8+ cells	-
Harris *et al.* (2012)	Conference abstract	416 ovarian cancer patients	Existing databank	-	-
**Combinations**					
de Lima *et al.* (2020)	Paper	3 ovarian cancer patients	Tumour biopsy, blood samples	TILs	-
MacGregor *et al.* (2019)	Paper	34 ovarian cancer patients	Tumour tissue, blood samples, TCGA databank	Immune cells, stromal cells and tumour cells	-
Zou *et al.* (2019)	Paper	Ovarian cancer cell lines	-	CAR-T cells	-
Tu *et al.* (2020)	Paper	Not mentioned	Oncomine, PrognoScan, K-M plotter and TCGA database	B cells, CD8+ T cells, CD4+ T cells, neutrophils, DCs and macrophages	-
Fucikova *et al.* (2019)	Paper	80 ovarian cancer patients<br>20 ovarian cancer patients	Tumour tissue	CD8+ T cells, DC-LAMP+ DCs and CD20+ B cells	CD8+T-cells: 31.3% (TIM-3)
		308 ovarian cancer patients	Existing database	-	-
Rådestad *et al.* (2018)	Paper	23 ovarian cancer patients	Tumor tissue, blood, ascites	Peripheral blood lymphocytes, tumour-associated lymphocytes and TILs	CD8+ T cells: 53.3% (PD-1+)<br>20.2% (TIM-3+)<br>CD4+ T cells: 41.8% (PD-1+)<br>15.9% (ANTI_+)

Imai *et al.* (2018)	Paper	54 ovarian cancer patients	Tumour tissue, ascites	CD4+ and CD8+ T cells	CD8+ T cells: 57.7% (PD-1)<br>5.0% (LAG-3)<br>4.9% (TIM-3)<br>15.7% (BTLA)<br>CD4+ T cells: 65.8% (PD-1)<br>10.6% (LAG-3)<br>4.3% (TIM-3)<br>37.6% (BTLA)
Huang *et al.* (2017)	Conference abstract	Murine model	-	TILs, TALs	-
Bergamini *et al.* (2019)	Conference abstract	34 ovarian cancer patients	Tumour tissue, blood and ascites	T cells	ITTCs: 61.9% (PD-1)<br>61.7% (LAG-3)<br>61.7% (TIM-3)
Kaufmann *et al.* (2019)	Conference abstract	-	Tumour tissue	TILs	-
Anderson *et al.* (2019)	Conference abstract	Ovarian cancer cell lines	-	-	-
De May *et al.* (2019)	Conference abstract	Ovarian cancer cell lines	-	CD8+ T cells	92% (CD49d)
Laeremans *et al.* (2018)	Conference abstract	Not mentioned	Tumour tissue	T cells	-
Morgado *et al.* (2018)	Conference abstract	225 ovarian cancer patients	Tumour tissue	T cells	-
Laeremans *et al.* (2018)	Conference abstract	30 ovarian cancer patients	Tumour tissue	-	-
Harms *et al.* (2017)	Conference abstract	30 ovarian cancer patients	Tumour tissue	TILs	-
Ziello *et al.* (2017)	Conference abstract	Not mentioned	Tumour tissue	TILs	-
Gaillard *et al.* (2017)	Conference abstract	8 ovarian cancer patients	Blood and ascites	CD8+ T cells	-

Rådestad *et al.* (2016)	Conference abstract	24 ovarian cancer patients	Metastatic tumour tissue, blood and ascites	T cells	Tumor: 9.4% (LAG-3)<br>62.6% (PD-1)<br>7.5% (TIM-3)<br>4.0% (CTLA-4)<br>Blood: 0.8% (LAG-3)<br>13.4% (PD-1)<br>0.1% (TIM-3)<br>0.7% (CTLA-4)
Peper *et al.* (2016)	Conference abstract	Not mentioned	Tumour tissue	TILs	-
Dai *et al.* (2015)	Conference abstract	Murine model	Intraperitoneal tumour nests	T cells	-

CTLA-4: Cytotoxic T-lymphocyte associated protein 4, LAG-3: Lymphocyte-activation gene 3, TIM-3: T-cell immunoglobulin domain and mucin domain 3, PD-1: Programmed cell death protein 1, TILs: Tumour-infiltrating lymphocytes, DC-LAMP: dendritic cell-lysosomal associated membrane protein, Th: T helper cell, Treg: Regulatory T-cell, TCGA: The Cancer Genome Atlas, PBMCs: Peripheral blood mononuclear cells

Supplementary Table S2: Fluorochrome-conjugated antibodies used in flow cytometry staining

**Target**	**Fluorochrome**	**Clone**	**Manufacturer**
B220	PE-Cy5	RA3-6B2	BioLegend
CD11c	PE-Cy7	HL3	BD Biosciences
CD11b	BV750	M1/70	BD Biosciences
	BV750	M1/70	BioLegend
CD134/OX-40	BV711	OX-86	BD Biosciences
CD19	BV711	1D3	BD Biosciences
CD223	BUV661	C9B7W	BD Biosciences
CD25	BB515	PC61	BD Biosciences
CD3e	PE-Cy7	17A2	BioLegend
	BUV737	145-2C11	BD Biosciences
CD366 (TIM-3)	BB515	5D12/TIM-3	BD Biosciences
CD44	BUV395	IM7	BD Biosciences
CD45	BUV805	30-F11	BD Biosciences
CD4	BV480	RM4-5	BD Biosciences
CD62L	BV785	MEL-14	BD Biosciences
CD64	PE	X54-5/7.1	BioLegend
CD69	APC-Cy7	H1.2F3	BioLegend
CD8a	BUV496	53-6.7	BD Biosciences
Granzyme B	APC-R700	GB11	BD Biosciences
IFN-γ	BB700	XMG1.2	BD Biosciences
Ki67	BV650	B56	BD Biosciences
Ly6C	APC-Cy7	AL-21	BD Biosciences
Ly6G	BV605	1A8	BD Biosciences
	BUV563	1A8	BD Biosciences
MHCII (IA/IE)	BUV395	2G9	BD Biosciences
NK1.1	BUV563	PK136	BD Biosciences
	BUV661	PK136	BD Biosciences
PD-L1/CD274	BUV615	MIH5	BD Biosciences

PD-1	PE-CF594	J43	BD Biosciences
Siglec-H	BB700	440c	BD Biosciences
Siglec-F	BV605	E50-2440	BD Biosciences
TCRγδ	BUV615	GL3	BD Biosciences
	BV650	GL3	BD Biosciences
F4/80	APC-R700	T45-2342	BD Biosciences
FoxP3	AF647	MF23	BD Biosciences
XCR1	BV421	ZET	BioLegend

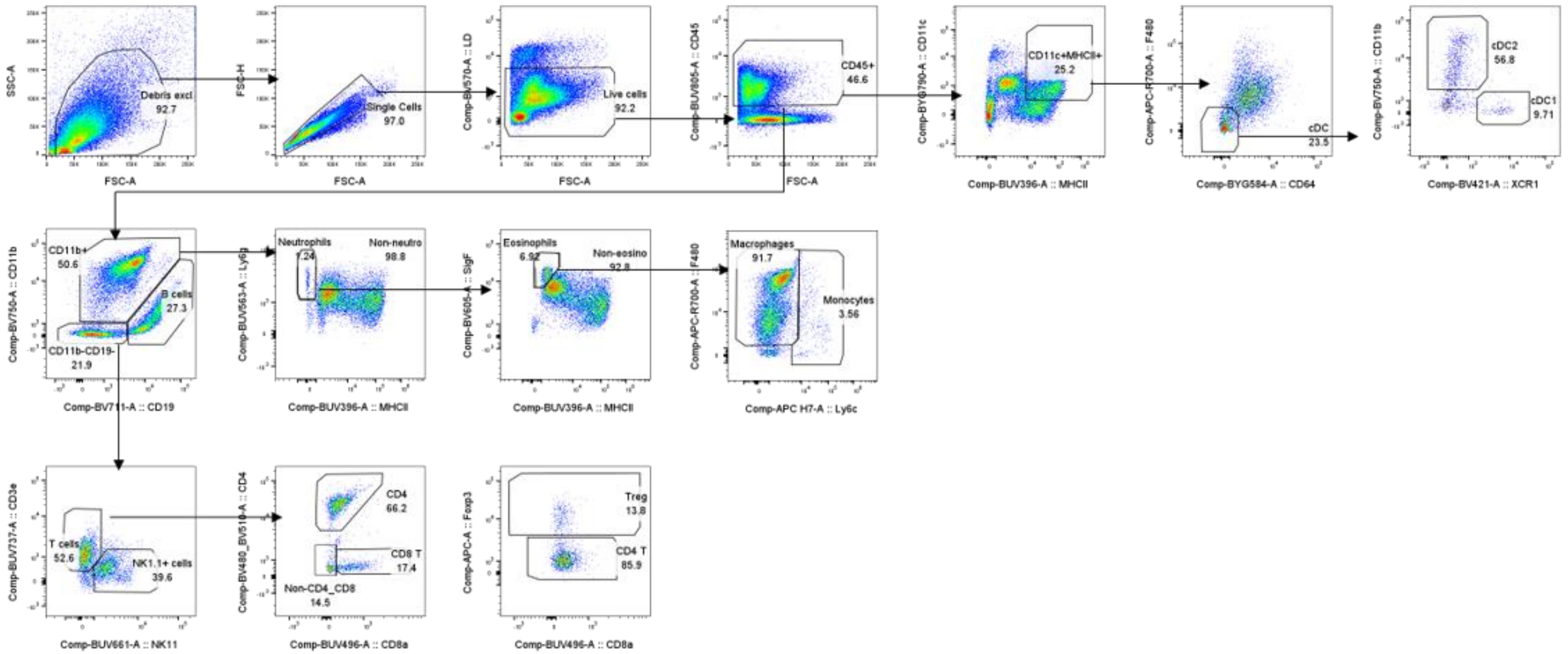

Supplementary Figure S1: Gating strategy of flow cytometry analysis general immune panel.

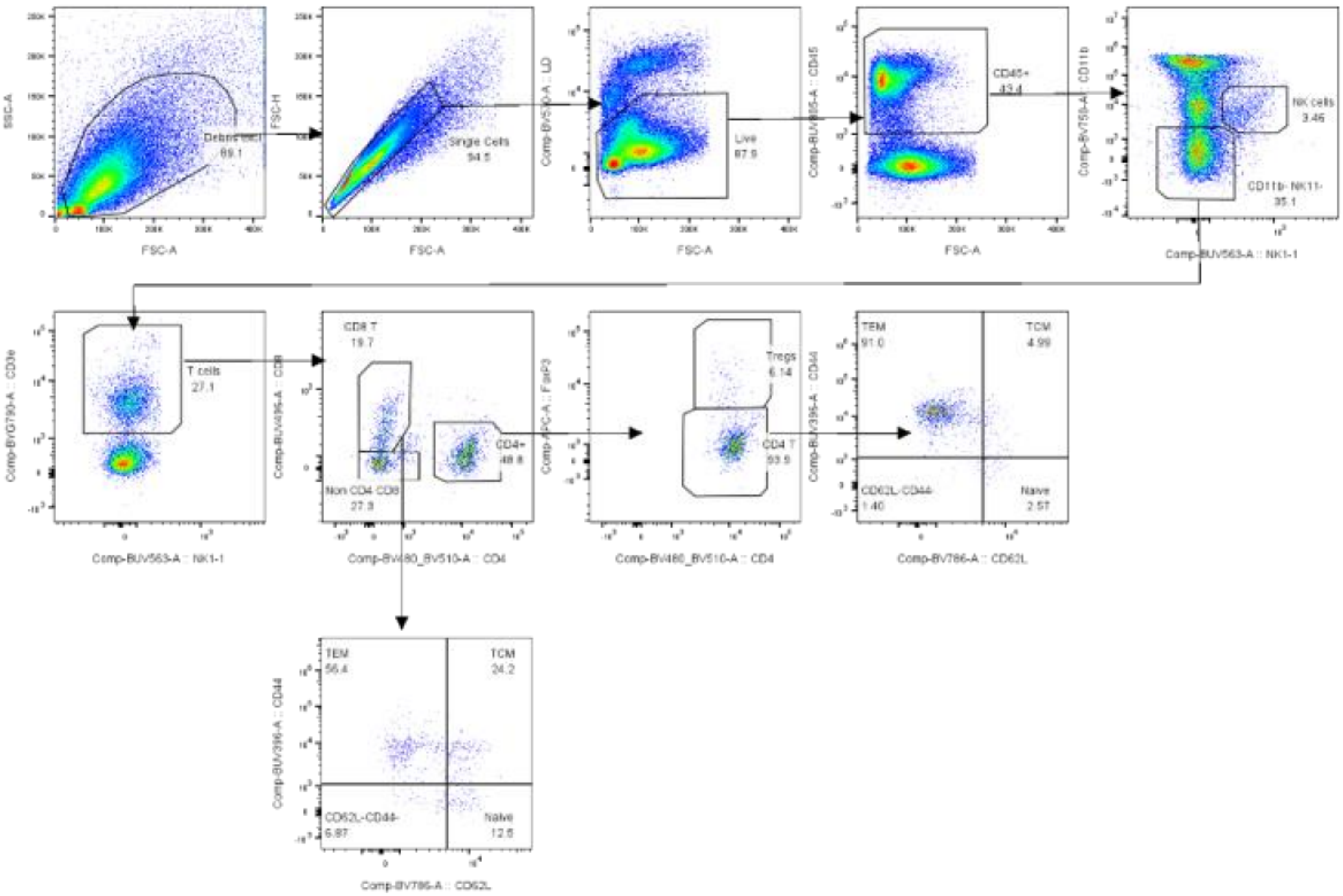

Supplementary Figure S2: Gating strategy of flow cytometry analysis -T-cell panel including activation and exhaustion markers.

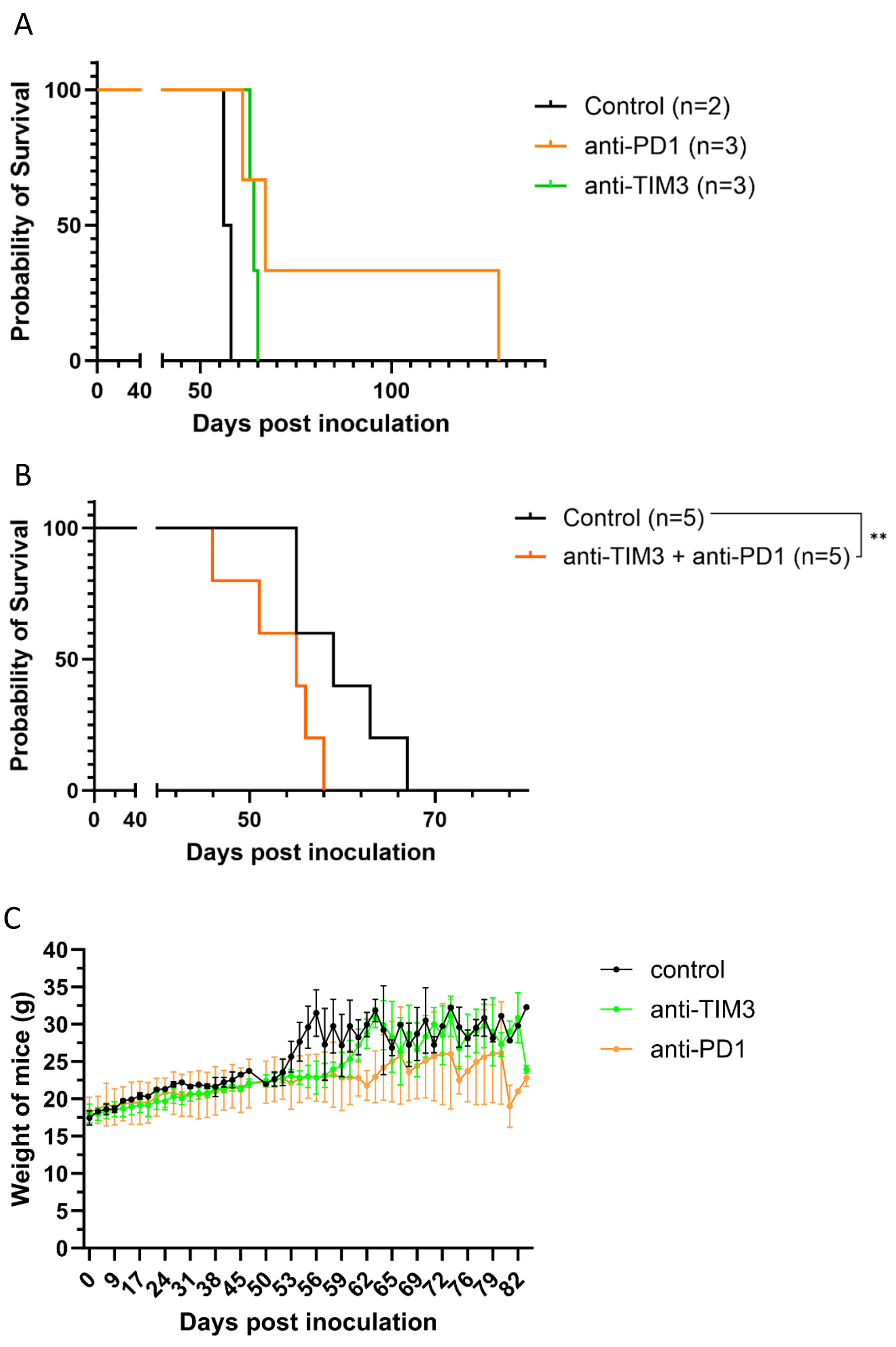

Supplementary Figure S3: Kaplan-Meier survival curve taking into account the ascites drainage at 32 grams of ID8-fLuc bearing mice following monotherapy (A) or combined immune checkpoint inhibition (B). (C) Scatter plot showing the mean weight of ID8-fLuc bearing mice following monotherapy. Mice were treated with either vehicle control injections with DPBS, anti-PD1 (50 µg, day 20/22/24/26/28) or anti-TIM3 (350 µg, sequential treatment scheme, day 20/24/27/31/34/38) or a combination of both immune checkpoint inhibitor treatments, intraperitoneally. **P-value < 0.01

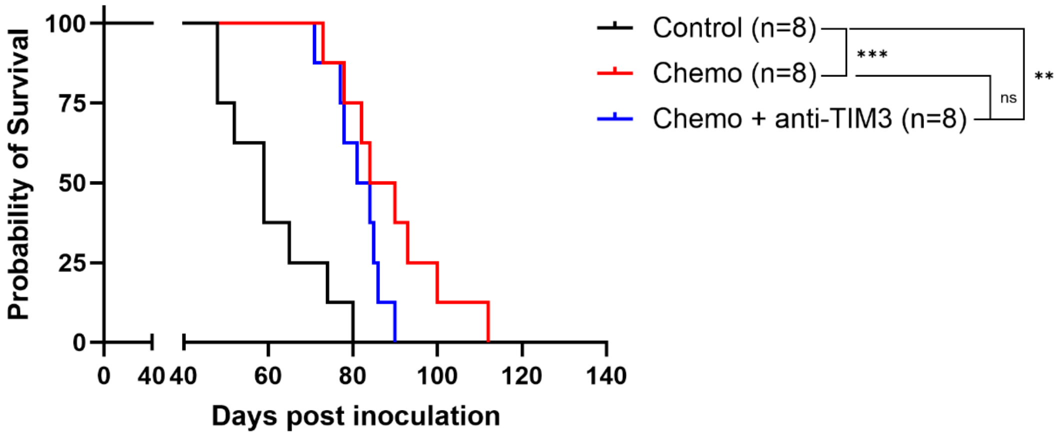

Supplementary Figure S4: Kaplan-Meier survival curve taking into account the ascites drainage at 32 grams of ID8-fLuc bearing mice following simultaneous administration of chemotherapy and anti-TIM3. All mice were treated with either vehicle control, chemotherapy consisting of carboplatin (100mg/m)g and paclitaxel (10mg/kg) or a combination of chemotherapy and anti-TIM3 ICI in the simultaneous treatment scheme (350 µg, day 20/24/27/31/34/38), through intraperitoneal injections. ***p-value <0.005, ns= not significant

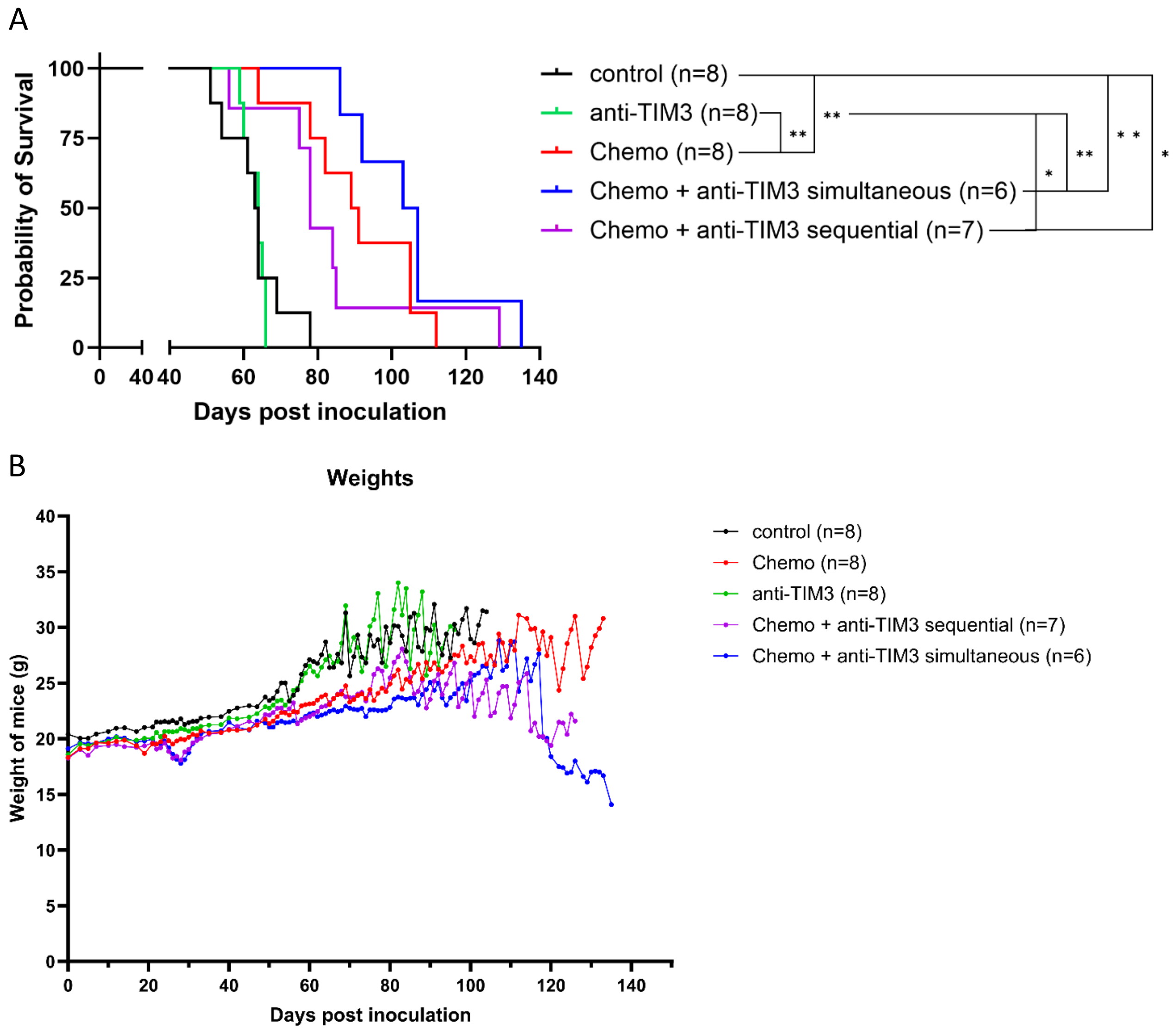

Supplementary Figure S5: (A) Kaplan-Meier survival curve on ID8-fLuc bearing mice following different combination administration schemes with chemotherapy and anti-TIM3 treatment. (B) Scatter plot showing the mean weight of ID8-fLuc bearing mice following different combination administration schemes with chemotherapy and anti-TIM3 treatment. Mice were treated with either vehicle control, anti-TIM3 (350 µg/injection) in monotherapy following a bi-weekly administration for four weeks (D28/32/35/39/42/46/49/53) or in combination with chemotherapy consisting of carboplatin (100 mg/kg) + paclitaxel (10 mg/kg) on day 21 with anti-TIM3 administered in either the sequential treatment schedule (D28/32/39/42/46/49/53) or a simultaneous administration scheme (D20/24/27/31/34/38/41/45). *p-value<0.05, **p-value<0.005

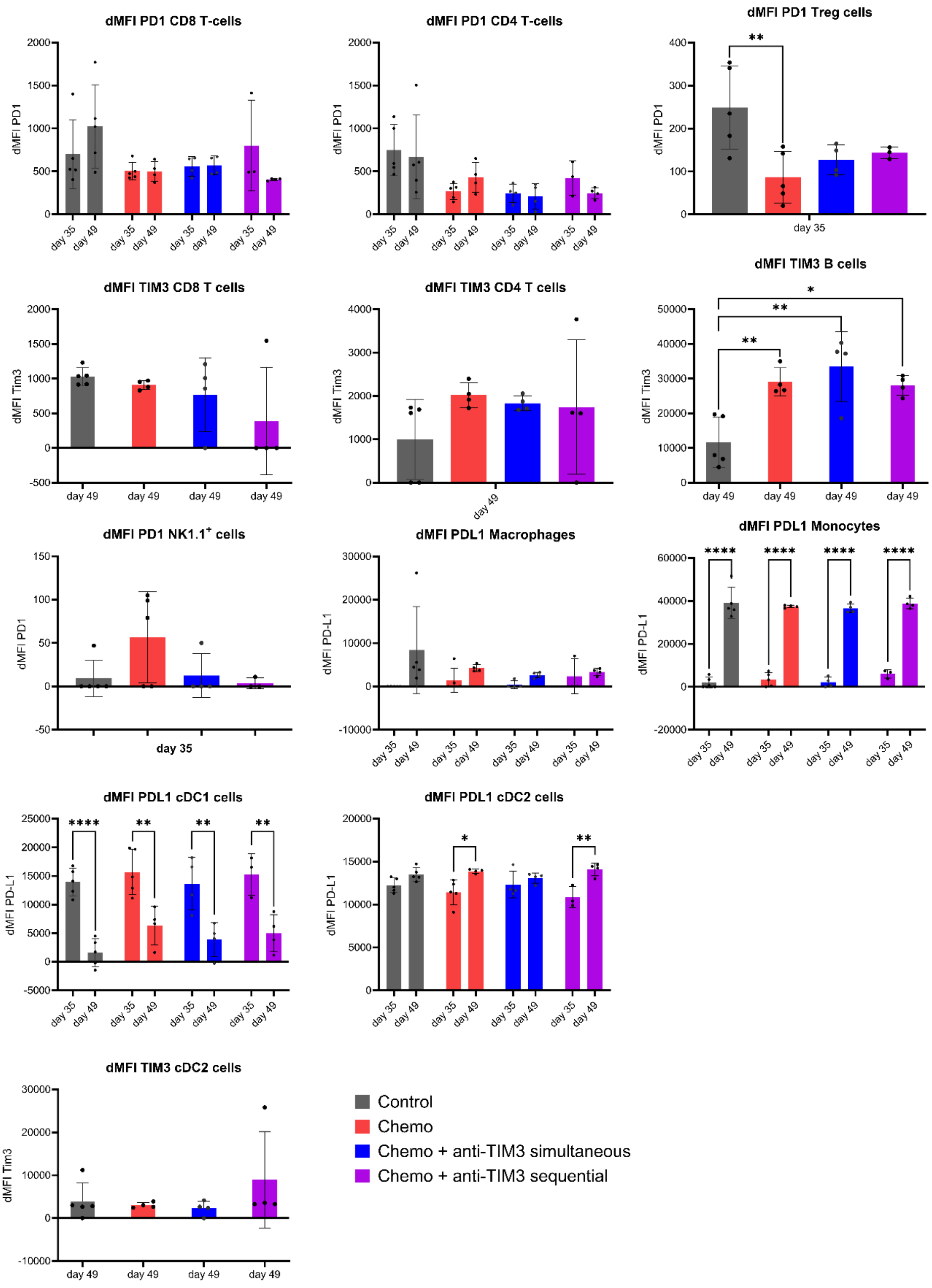

Supplementary Figure S6: flow cytometry analysis of immune cells isolated from peritoneal washings of ovarian cancer bearing mice. Treg: regulatory T-cell, cDC1/2: conventional dendritic cell 1/2, NK1.1^{+} cells: natural killer cells 1.1, PD1: programmed cell death protein 1, PD-L1: programmed death-ligand 1, TIM3: T-cell immunoglobulin and mucin domain 3, dMFI: difference in mean fluorescence intensity (MFI) between population and FMO (fluorescence minus one) control *p-value < 0.05, **p-value <0.005

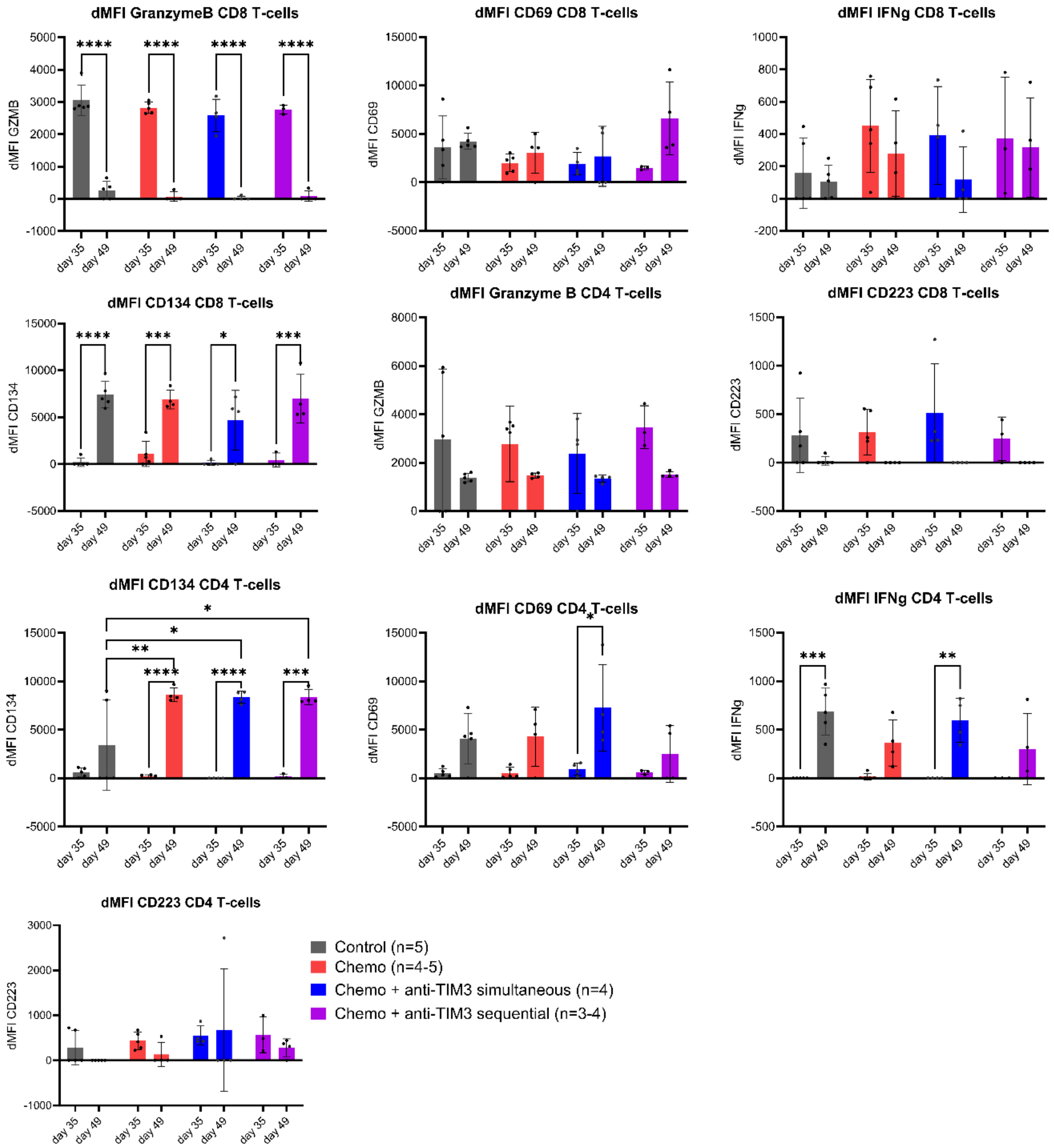

Supplementary Figure S7: activation and exhaustion markers on T-cells isolated from peritoneal washings of ovarian cancer bearing mice. IFNg: interferon-gamma, dMFI: difference in mean fluorescence intensity (MFI) between population and FMO (fluorescence minus one) control *p-value < 0.05, **p-value <0.005

	CD8⁺ T cells	*CD20⁺ B cells*	*CD4⁺ T cells*
Control	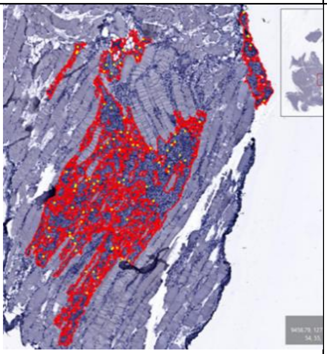	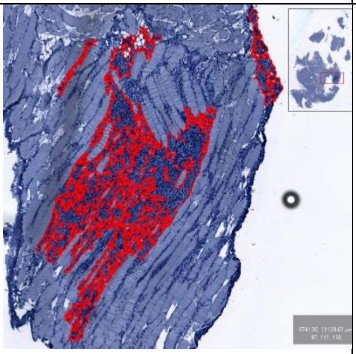	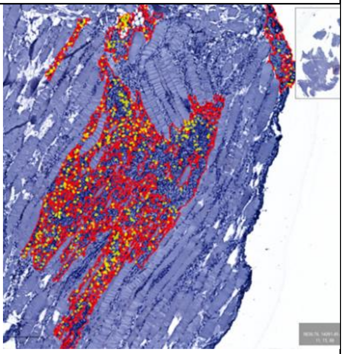
Chemo	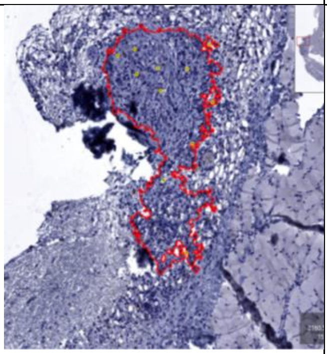	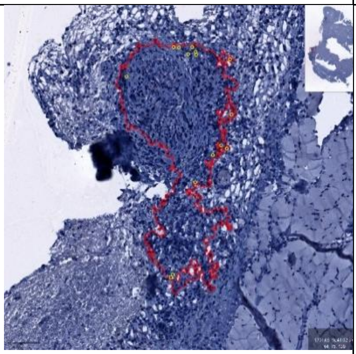	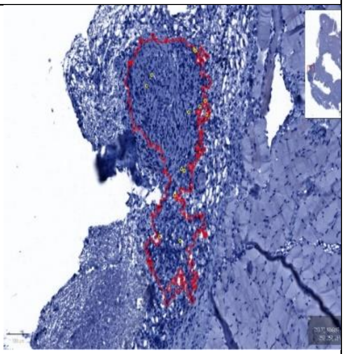
Chemo + anti-TIM3 simultaneous	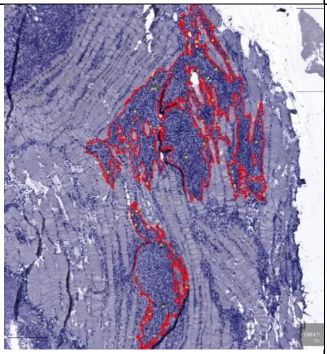	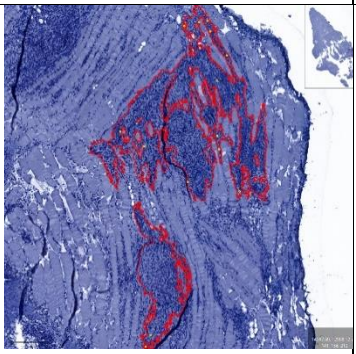	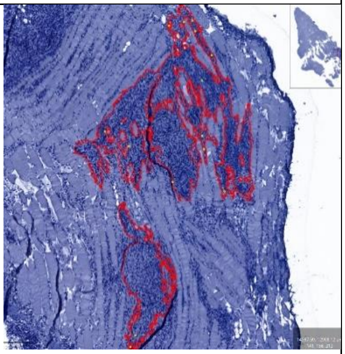
Chemo + anti-TIM3 sequential	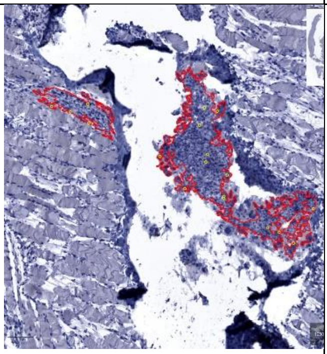	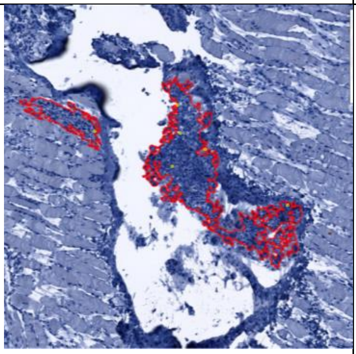	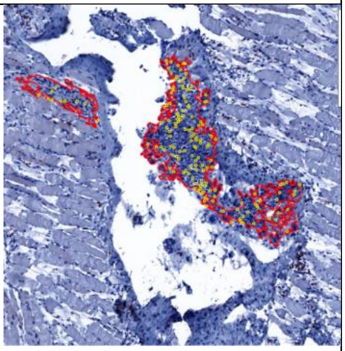

Supplementary Figure S8: Images of immunohistochemical analysis performed using QuPath 0.4.4. on peritoneal tumor biopsies of mice sacrificed on day 35 post inoculation.

	CD8⁺ T cells	*CD20⁺ B cells*	*CD4⁺ T cells*
Control	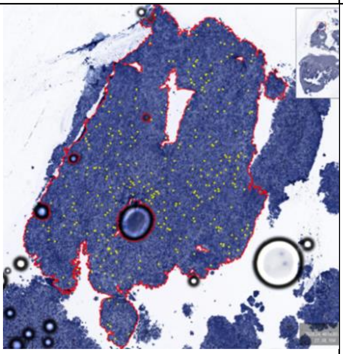	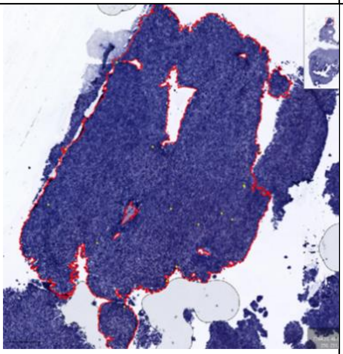	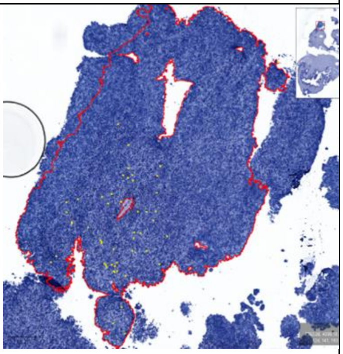
Chemo	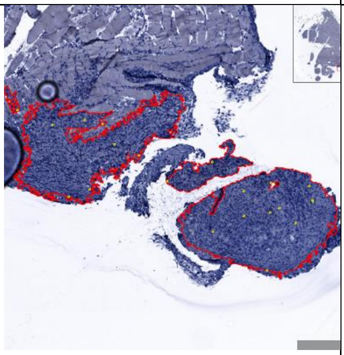	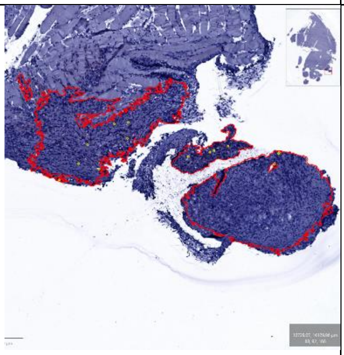	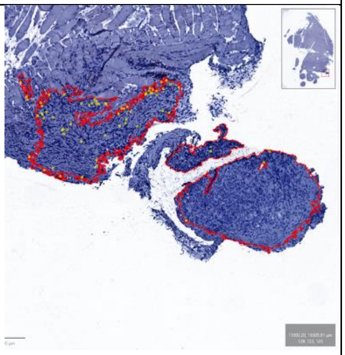
Chemo + anti-TIM3 simultaneous	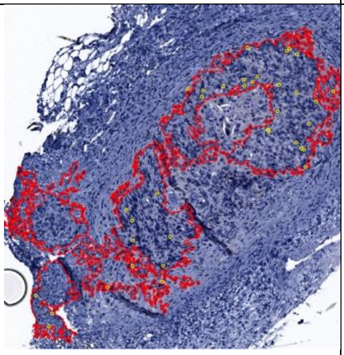	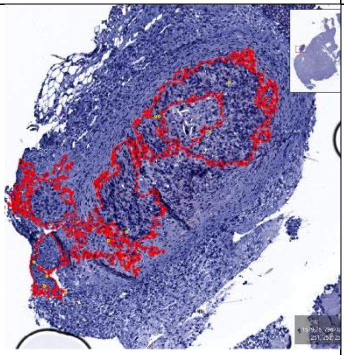	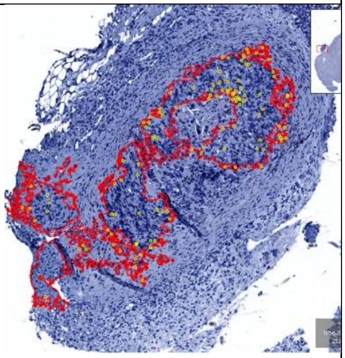
Chemo + anti-TIM3 sequential	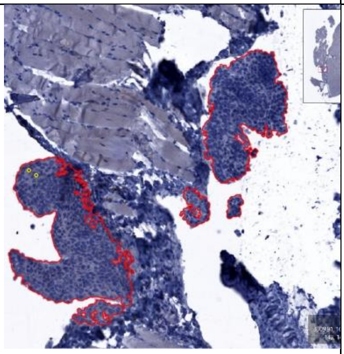	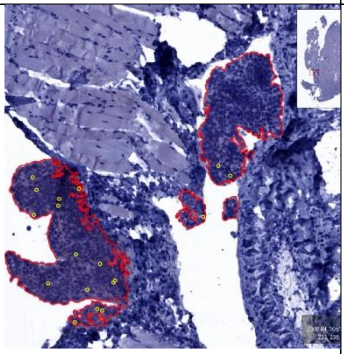	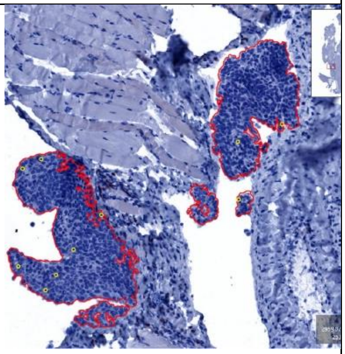

Supplementary Figure S9: Images of immunohistochemical analysis performed using QuPath 0.4.4. on peritoneal tumor biopsies of mice sacrificed on day 49 post inoculation.
